# Optimal Isolation Protocols for Examining and Interrogating Mononuclear Phagocytes From Human Intestinal Tissue

**DOI:** 10.3389/fimmu.2021.727952

**Published:** 2021-09-01

**Authors:** Chloe M. Doyle, Erica E. Vine, Kirstie M. Bertram, Heeva Baharlou, Jake W. Rhodes, Suat Dervish, Martijn P. Gosselink, Angelina Di Re, Geoffrey P. Collins, Faizur Reza, James W. T. Toh, Nimalan Pathma-Nathan, Golo Ahlenstiel, Grahame Ctercteko, Anthony L. Cunningham, Andrew N. Harman, Scott N. Byrne

**Affiliations:** ^1^Centre for Immunology and Allergy Research, The Westmead Institute for Medical Research, Westmead, NSW, Australia; ^2^Centre for Virus Research, The Westmead Institute for Medical Research, Westmead, NSW, Australia; ^3^School of Medical Sciences, Faculty of Medicine and Health, The University of Sydney, Westmead, NSW, Australia; ^4^Westmead Clinical School, Faculty of Medicine and Health, The University of Sydney, Westmead, NSW, Australia; ^5^Westmead Cytometry, The Westmead Institute for Medical Research, Westmead, NSW, Australia; ^6^Department of Colorectal Surgery, Westmead Hospital, Westmead, NSW, Australia; ^7^Storr Liver Centre, The Westmead Institute for Medical Research, Westmead, NSW, Australia; ^8^Blacktown Clinical School, Western Sydney University, Blacktown, NSW, Australia; ^9^Blacktown Hospital, Western Sydney Local Area Health District (WSLHD), Blacktown, NSW, Australia

**Keywords:** dendritic cells (DC), flow cytometry, human tissue, intestine, enzymatic digestion, macrophage – cell, mononuclear phagocyte cells (MNP)

## Abstract

The human intestine contains numerous mononuclear phagocytes (MNP), including subsets of conventional dendritic cells (cDC), macrophages (Mf) and monocytes, each playing their own unique role within the intestinal immune system and homeostasis. The ability to isolate and interrogate MNPs from fresh human tissue is crucial if we are to understand the role of these cells in homeostasis, disease settings and immunotherapies. However, liberating these cells from tissue is problematic as many of the key surface identification markers they express are susceptible to enzymatic cleavage and they are highly susceptible to cell death. In addition, the extraction process triggers immunological activation/maturation which alters their functional phenotype. Identifying the evolving, complex and highly heterogenous repertoire of MNPs by flow cytometry therefore requires careful selection of digestive enzyme blends that liberate viable cells and preserve recognition epitopes involving careful selection of antibody clones to enable analysis and sorting for functional assays. Here we describe a method for the anatomical separation of mucosa and submucosa as well as isolating lymphoid follicles from human jejunum, ileum and colon. We also describe in detail the optimised enzyme digestion methods needed to acquire functionally immature and biologically functional intestinal MNPs. A comprehensive list of screened antibody clones is also presented which allows for the development of high parameter flow cytometry panels to discriminate all currently identified human tissue MNP subsets including pDCs, cDC1, cDC2 (langerin^+^ and langerin^-^), newly described DC3, monocytes, Mf1, Mf2, Mf3 and Mf4. We also present a novel method to account for autofluorescent signal from tissue macrophages. Finally, we demonstrate that these methods can successfully be used to sort functional, immature intestinal DCs that can be used for functional assays such as cytokine production assays.

## Introduction

Intestinal mononuclear phagocytes (MNPs), specifically dendritic cells (DC), macrophages and monocytes, play a major role in maintaining tolerance to food-derived antigens and resident microbiota without compromising the hosts ability to respond to invading pathogens (1). DCs are professional antigen presenting cells (APC) capable of antigen uptake, processing and migration out of the tissue to draining lymph nodes where they present antigens and activate naïve T cells (2, 3). In contrast, macrophages perform this antigen presenting function weakly and do not readily migrate out of the tissue. Their primary function is to phagocytose and destroy invading pathogens as well as secrete a variety of immune-modulating cytokines (2, 3). Monocytes migrate into tissue from blood and differentiate into monocyte-derived macrophages or DCs. Embedded within these broad cell types are a diverse range of DC and macrophage subsets that each possess their own tissue-specific phenotype and function. Accurate identification of these subsets in the human intestine is required before we can understand their role in homeostasis and disease settings. Indeed, the misidentification of MNP subsets has caused much confusion in the literature (4–8).

Isolating MNPs from human tissues is a challenging task as the techniques used to liberate them can alter their phenotype and affect their viability (9). We have previously published optimised methods for isolating and interrogating immature DCs and macrophages from human abdominal skin *via* enzymatic digestion (9). However, skin and intestinal tissue have marked phenotypic, functional and structural differences which necessitates a modified approach to isolating MNPs. Recent literature has described a suite of consistent markers used to define all currently known subsets of human tissue DCs and macrophages (8–13). In human abdominal skin, these comprise XCR1^+^ cDC1 (conventional DC), CD1c^+^ cDC2 that includes langerin expressing and langerin negative populations and CD14-expressing cells including tissue-resident autofluorescent (AF) macrophages and monocyte-derived macrophages (13). In human intestinal tissue, three populations of DCs have been identified using CD103 and SIRP*α* (11, 12) and four intestinal CD14^+^ macrophage populations (Mf1-4) have been identified using HLA-DR, CD14, CD11c and CD11b (10). More recently, high-resolution analyses have revealed a subpopulation within human blood cDC2s (CD1c^+^ DCs) that expresses CD14 and a monocyte-like gene signature termed DC3s (14, 15). As defined by the literature, DC3s are CD88^-^ CD1c^+^ CD163^+^ and express varying levels of CD14 (14, 15).

In this study, we present an intestinal tissue specific MNP isolation protocol to liberate high yields of viable, immature and biologically active MNPs from human intestinal jejunum, ileum and colon as well as terminal ileum biopsies. We also present techniques to anatomically separate the mucosa and submucosa, including their associated lymphoid follicles being Peyer’s Patches in the small bowel and lymphoid aggregates in the large bowel, to better understand these distinct immune compartments. We emphasize the importance of carefully selecting antibodies that target the appropriate epitope post-digestion as well as markers that accurately define intestinal-derived MNPs according to the most recent and reliable literature. Further, we present a high-parameter flow cytometry gating strategy to identify all currently known human MNPs in human tissues. We also include a method for correcting AF spillover from tissue-resident macrophages which considerably improves the accuracy of measuring cell surface expression levels and correct MNP definition.

## Methods

### Human Specimens

This study was approved by the Western Sydney Local Area Health District (WSLHD) Human Research Ethics Committee (HREC); reference number (4192) AU RED HREC/15 WMEAD/11. Large human intestinal specimens were taken with informed consent from patients undergoing surgery for intestinal cancer, 10-20 cm away from tumours, where present. Samples were processed within 2 hours of collection except for samples destined for cell sorting which were covered in Roswell Park Memorial Institute (RPMI) (Lonza, Switzerland) 1640 supplemented with 0.25% gentamycin and stored overnight at 4°C for processing the following morning.

### Tissue Processing

Typical whole tissue intestinal specimens ranged in size from 5-40cm^2^, with all data obtained with whole tissue specimens unless biopsies are stated. The muscularis externa was mechanically removed from the submucosa using curved surgical scissors and forceps. The tissue was then cut into approximately 25mm^2^ pieces and incubated for 15 minutes twice in RPMI-1640 (Lonza) supplemented with 10% Foetal Bovine Serum (FBS) (Sigma-Aldrich, Missouri, USA), 0.3% Dithiothreitol (DTT) (Sigma-Aldrich) and 2mM EDTA at 37°C (herein referred to as DTT treatment). The tissue was washed in Dulbecco’s Phosphate Buffered Saline Mg^++^/Ca^++^ free (DPBS) (Lonza) prior to incubation on a MACSmix Tube Rotator (Miltenyi Biotec, California, USA) 2-3 times in RPMI-1640 supplemented with 5*µ*L/mL DNase I (Worthington Industries, New Jersey, USA) and 3mg/mL collagenase type IV (Worthington) for 30 min each time at 37°C. The supernatant was separated from undigested intestinal tissue using a tea strainer. The cell suspension was passed through a 100*µ*M cell strainer (Greiner Bio-One, North Carolina, USA) before being washed twice in DPBS. The single-cell suspension in RPMI-1640 was spun on a Ficoll-Paque PLUS (GE Healthcare, Illinois, USA) gradient and mononuclear cells were harvested from the RPMI-Ficoll interface. When required, the mucosa was anatomically separated from the submucosa using a dissection microscope and forceps. Lymphoid follicles present in the mucosa and submucosa were then dissected out using a scalpel. The mucosa was digested as above, with the submucosa not requiring the DTT treatment. For terminal ileum biopsies, a 10 min DTT treatment at 37°C was required. A single cell suspension from terminal ileum biopsies and isolated lymphoid follicles was generated by shaking them at 170rpm in a 37°C incubator in RPMI-1640 supplemented with 5*µ*L/mL DNase I and 3mg/mL collagenase type IV for 45 min.

### Flow Cytometry

Cells were labelled in aliquots of 1-3 x 10^6^ cells per 100*µ*L of DPBS. Non-viable cells were excluded by staining with Fixable Viability Stain 700 (BD Biosciences, New Jersey, USA). Cells were washed with FACS wash (1% FBS (v/v), 2 mM EDTA, 0.1% sodium azide (w/v) in PBS) and surface stained with the antibodies indicated in **Table 1** in 10*µ*L Brilliant stain buffer plus (BD Biosciences) for 30 mins on ice. Cells were washed twice with FACS wash prior to resuspension in 100*µ*L of CytoFix (BD Biosciences) for 15 mins at 4°C. Cells were washed and resuspended in 100*µ*L of FACS wash. Flow cytometry was performed on an LSR Fortessa or FACSymphony flow cytometers (BD Biosciences) with BD Diva Software (V8.0) and data analysed on FlowJo (TreeStar V10.7.1).

**Table 1:** FACSymphony mononuclear phagocyte phenotyping panel.

Marker	Company	Clone	Fluorophore	Purpose	Concentration (*µL*/100*µL*)
**Viability**	BD Biosciences	–	FVS700	Live cells	1:10, 000
**HLA-DR**	BD Biosciences	G46-6	BUV395	Myeloid cells	0.5
**CD45**	BD Biosciences	HI30	BB755	Immune cells	0.5
**CD3**	BD Biosciences	UCTH1	BUV496	T lymphocytes	5
**CD19**	BioLegend	HIB19	BV750	B lymphocytes	5
**CD14**	BD Biosciences	M5E2	BUV737	Macrophages	2.5
**Calprotectin**	Invitrogen	MAC387	PE	Infiltrating monocytes	2.5
**CD88**	BioLegend	S5/1	PE Dazzle 594	Monocytes	0.5
**CD1c**	BD Biosciences	F10/21A3	BUV805	cDC2	2.5
**FC*ϵ*R1*α***	Novus Bio	9E1	AF488	cDC2	1.5
**FC*ϵ*R1*α***	BD Biosciences	AER-37	APC	cDC2	5
**CD11b**	BD Biosciences	ICRF44	BV711	MNPs	2
**CD11c**	BD Biosciences	B-Ly6	BB515	MNPs	1.5
**CD123**	BioLegend	6H6	PE/Cy5	pDCs	0.5
**CD163**	BioLegend	GHI/61	BV605	DC3	2.5
**SIRPα**	BioLegend	SE5A5	APC/Fire 750	DCs	2.5
**Langerin**	Miltenyi	MB22-9F5	PE-Vio770	cDC2	1.5
**XCR1**	BioLegend	S15046E	BV421	cDC1	4
**CD141**	Miltenyi	AD5-14H2	APC	cDC1	2.5

### Fluorescence Activated Cell Sorting

Single-cell suspensions to be sorted were enriched for CD45^+^ cells using anti-CD45 microbeads (Miltenyi Biotec) as per the manufacturer’s instructions. CD45^+^ cells were stained with Live/Dead Near IR dead cell stain kit (Life Technologies, California, USA) in DPBS for 30 mins on ice, and washed with magnetic-activated cell sorting (MACS) wash [1% FBS (v/v), 2 mM EDTA in DPBS] before surface staining with antibodies indicated in **Table 2**. Cells were washed twice with MACS wash and filtered prior to sorting on a BD FACS Aria (130*µ*M nozzle) (BD Biosciences), with sorted cells collected into 1.5 ml Eppendorf tubes (Sigma-Aldrich) containing RPMI-1640 supplemented with 10*µ*M HEPES (Gibco, Massachusetts, USA), non-essential amino acids (Gibco), 1mM sodium pyruvate (Gibco) 50*µ*M 2-mercaptoethanol (Gibco), 10*µ*g/mL gentamycin (Gibco) and 10% (v/v) FBS (herein referred to as DC culture media).

**Table 2:** FACS Aria sort panel.

Marker	Company	Clone	Fluorophore	Purpose	Concentration (*µL*/100*µL*)
**Viability**	ThermoFisher	–	NIR	Live cells	1: 500
**HLA-DR**	Miltenyi	G46-6	PerCP	Myeloid cells	2
**CD45**	BD Biosciences	HI30	BV786	Immune cells	1
**CD3**	Miltenyi	REA613	APC-Vio770	T lymphocytes	2.5
**CD19**	Miltenyi	REA674	APC-Vio770	B lymphocytes	1
**CD14**	BD Biosciences	M5E2	BV421	Macrophages	2.5
**CD1c**	BD Biosciences	F10/21A3	PE	cDC2	2
**CD11c**	BD Biosciences	B-Ly6	PE-CF594	MNPs	1.5
**Langerin**	Miltenyi	MB22-9F5	PE-Vio770	cDC2	1.5
**CD123**	BD Biosciences	9F5	BV711	pDCs	1
**XCR1**	BioLegend	S15046E	APC	cDC1	4

### Culturing of *Ex Vivo* Mononuclear Phagocytes

Following dead cell depletion (STEMCELL Technologies, Vancouver, Canada) as per the manufacturer’s instructions and CD45^+^ enrichment, 5 x 10^5^ cells per condition were taken as a time 0-hour aliquot. All remaining cells were cultured at 1 x 10^6^ cells/mL for 14 hours in DC culture media. Cells were then washed once in DPBS and stained with Fixable Viability Stain 700. Cells were washed with FACS wash and surface stained with antibodies indicated in **Table 1**, with drop-in CD54-PE (clone: HA58; 2.5*µ*L/100*µ*L), CD80-PE (clone: L307.4; 3*µ*L/100*µ*L), CD83-APC (clone: HB15e; 2.5*µ*L/100*µ*L) and CD86-APC (clone: 2331 (FUN-1); 2.5*µ*L/100*µ*L). Cells were washed twice with FACS wash prior to resuspension in 100*µ*L of CytoFix (BD Biosciences) for 15 mins at 4°C. Cells were washed and resuspended in 100*µ*L of FACS wash for acquisition.

### Intracellular Cytokine Staining

Sorted intestinal-derived MNPs were cultured in DC culture media and stimulated with 1*µ*g/mL of the TLR7/8 ligand, R848 (InvivoGen, California, USA). After 2 hours, 2.5*µ*g/mL Brefeldin A (Sigma-Aldrich) was added to prevent cytokine secretion into the supernatant. After a total of 16 hours, cells were washed with DPBS and stained with Live/Dead Near IR dead cell stain kit. Cells were washed with FACS wash and resuspended in 100*µ*L of CytoFix/CytoPerm (BD Biosciences) for 15 minutes at 22°C. Cells were washed twice with Perm Wash (1% FCS (v/v), 1% BSA (w/v), 0.1% saponin (w/v), 0.1% sodium azide (w/v) in PBS) and stained with anti-IL-6 APC (clone: MQ2-13A5; 2.5*µ*L/100*µ*L), anti-IL-23p40 PE (clone: C8.6; 0.25*µ*L/100*µ*L) and anti-TNF BV650 (clone: MAB11; 2.5*µ*L/100*µ*L) for 30 minutes at 22°C. Cells were washed and resuspended in 100*µ*L of FACS wash prior to acquisition.

### Statistical Analysis

Statistical analysis was conducted using GraphPad Prism v9.1.2 (San Diego, CA). A Student’s paired t test (comparing 2 groups) or a Kruskal Wallis test with Dunn’s multiple comparisons test (comparing more than 2 groups) was used.

## Results

### Optimisation of Enzymatic Digestion Protocols for the Isolation and Interrogation of Intestinal Mononuclear Cells

**Figure 1:**
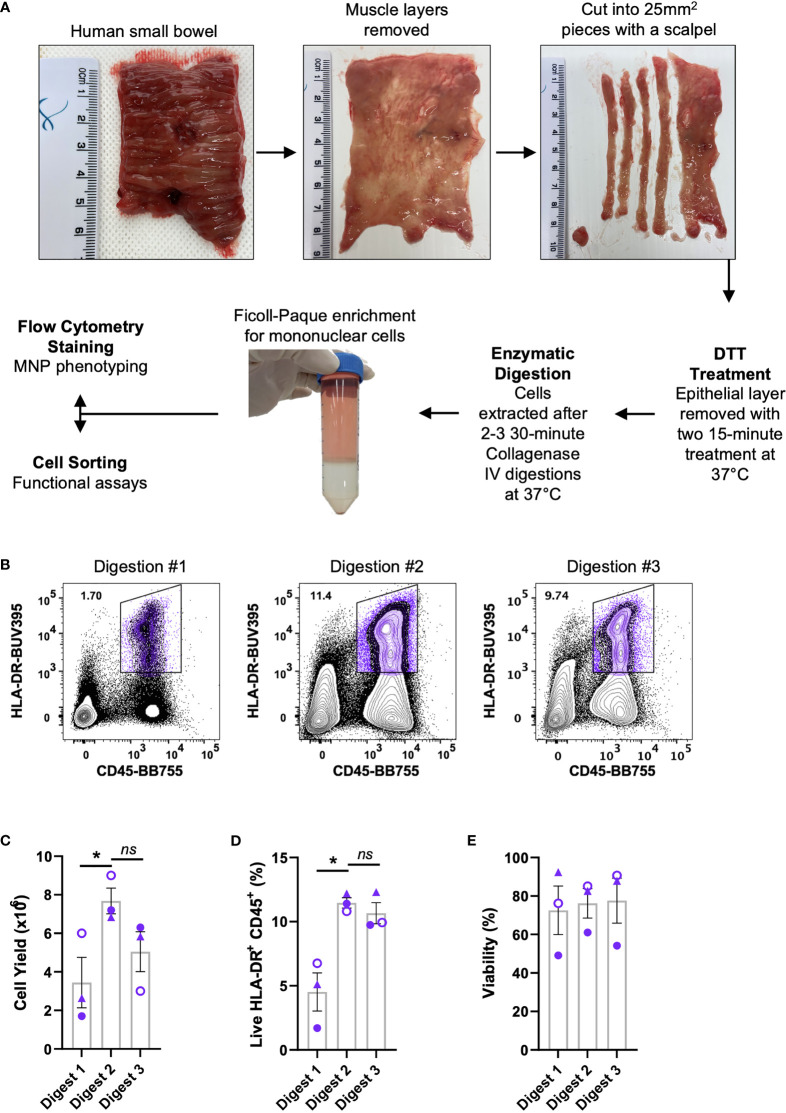
Isolation of immune cells from human intestinal tissues. Discarded human intestinal tissues were obtained within one hour of surgery. **(A)** Underlying tissue was removed using curved surgical scissors before being diced into small pieces with a scalpel. Tissue was treated with DTT for two 15 min incubations at 37°C prior to 2-3 30 min digestions with collagenase type IV. Mononuclear cells were enriched using a Ficoll-Paque gradient before cells were stained for phenotyping or cell sorting. **(B)** Representative plot of live HLA-DR^+^ CD45^+^ proportions from each digestion. **(C)** Cell yields from each digestion, counted post-Ficoll on a haemocytometer. **(D)** Percentage of live HLA-DR^+^ CD45^+^ cells from total cells as determined by flow cytometry. **(E)** Percentage of viability of each digestion as determined by flow cytometry with mean ± SEM. Each symbol represents an individual donor (n=3). Statistics was by a Kruskal Wallis test with Dunn’s multiple comparisons test comparing the sequential digests (*p < 0.05, ns, not significant).

We optimised a protocol for the mechanical separation and enzymatic digestion of human intestinal tissue to liberate MNPs. Enzymatic access to the mucosal tissue was increased by removing the underlying muscle layer and fine dicing the tissue with a scalpel. The epithelium and any remnant mucus were removed with DTT treatment prior to digestion with collagenase type IV at 37°C which we previously showed to the best collagenase blend for MNP tissue isolation (9) (**Figure 1A**). Using this method, we compared the yield and proportions of MNPs (CD3^-^ CD19^-^ HLA-DR^+^ CD45^+^) from sequential 30-minute collagenase digestions (**Figure 1B**). Digestions were performed in sequence rather than one extended digestion to both limit the exposure of the liberated cells to the enzyme, and to replenish the enzyme for optimal activity. The first digestion resulted in significantly lower yield and proportion of MNPs than the subsequent digestions, suggesting that 30 minutes was not long enough to liberate our cells of interest from whole tissue specimens. The second digestion provided the highest proportion of MNPs as well as the highest yield, in numbers suitable for phenotyping assays. (**Figures 1C, D**). A third digestion liberated additional cells which increased the total yield (**Figure 1C**), without compromising the proportion of HLA-DR^+^ CD45^+^ cells (**Figure 1D**) or their viability (**Figure 1E**). Two digestions were performed for phenotyping purposes, while three digestions were utilised when higher numbers of MNPs were required for cell sorting and functional assays.

### Manual Separation of Intestinal Tissue Compartments

**Figure 2:**
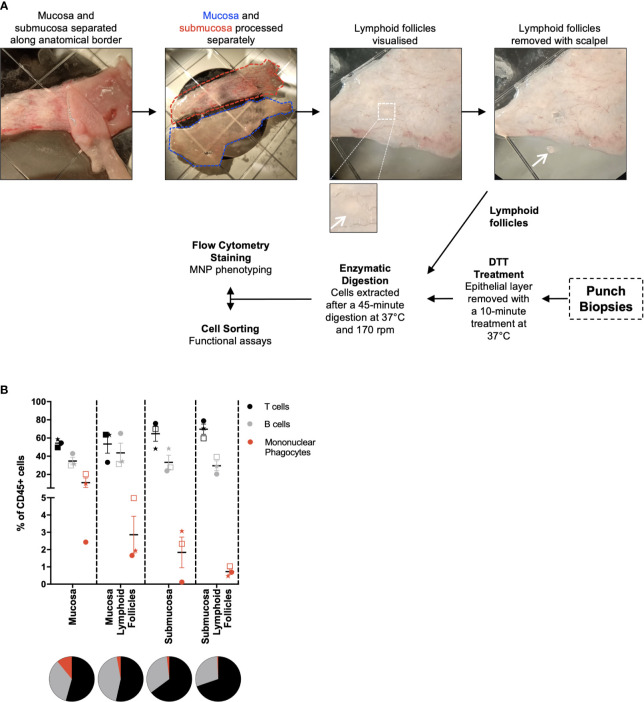
Separation of tissue compartments from intestinal tissue. Discarded human intestinal tissues were obtained and underlying tissue removed as in **Figure 1**. **(A)** Under a dissecting microscope, the mucosa and submucosa were mechanically separated with forceps. Mucosa and submucosa were then processed separately for the removal of lymphoid follicles. Insert: magnified view of a lymphoid follicle. Follicles are visualised and removed from each tissue layer using a scalpel. Mucosa and submucosa were processed as per **Figure 1**, with no DTT treatment for the submucosa. Lymphoid follicles were digested for 45 mins at 37°C at 170 rpm. When obtained, punch biopsies were DTT-treated for 10 mins at 37°C, before being digested as per the lymphoid follicles. **(B)** Cells liberated from tissue compartments were analysed by flow cytometry. CD3^+^ T cells, CD19^+^ B cells and HLA-DR^+^ mononuclear phagocytes were represented as a percentage of CD45^+^ cells with mean ± SEM. Each symbol represents an individual donor (n=3). Pie charts represent the mean as parts of whole.

Intestinal mucosal tissue consists of multiple compartments that require separation to investigate their unique immune cell profiles (16). To achieve this, the mucosa and submucosa were mechanically separated along the biological border of the two layers, the muscularis mucosa, and lymphoid follicles were isolated prior to digestion (**Figure 2A**). This was performed under light microscopy without the need for tissue staining, with lymphoid follicles being visually identified and removed with a scalpel. The mucosa was digested as per the protocol for whole tissue (**Figure 1A**), with the submucosa digested without DTT treatment as it does not have an epithelial layer. Lymphoid follicles (<2mm^2^), like biopsies (<5mm^2^), are smaller than the diced pieces of whole tissue (25mm^2^) and as such were fully digested in a shorter, single digestion. The lymphoid follicles as well as terminal ileum biopsies underwent collagenase type IV digestion for 45 min at 37°, with biopsies receiving a 10 min DTT treatment at 37°C before digestion as described in **Figure 2A**. Using tissue from 3 individual donors and a minimal staining panel we were able to show by flow cytometry that of the CD45^+^ cells liberated, CD3^+^ T cells, CD19^+^ B cells and HLA-DR^+^ MNPs were represented in different proportions in each compartment (**Figure 2B**), confirming that we can manually dissect intestinal tissue to enrich for specific tissue compartments using visual confirmation.

### Accurate Identification of Tissue Dendritic Cell Subsets *via* Flow Cytometry

**Figure 3:**
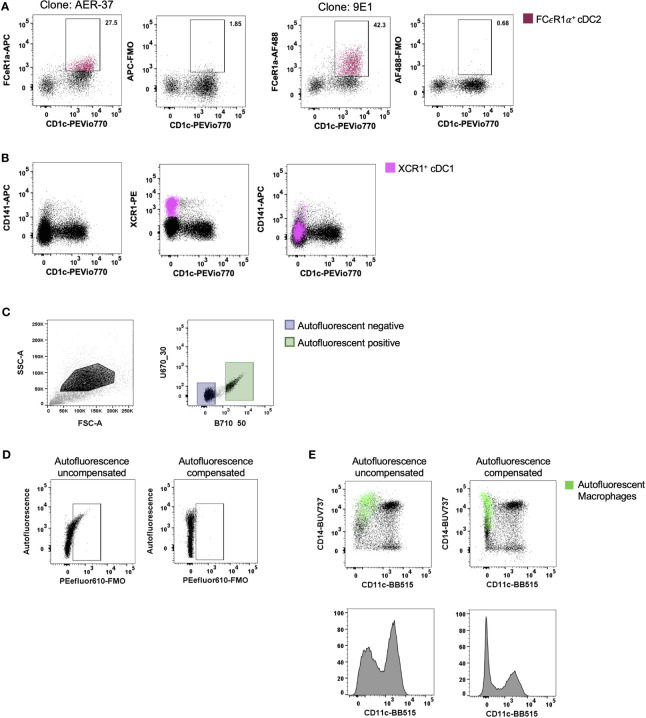
Optimisation of staining and acquisition parameters for flow cytometry. Intestinal mononuclear phagocytes were isolated as per **Figure 1**. **(A, B)** Cells were stained to isolate HLA-DR^+^ CD45^+^ CD3^-^ CD19^-^ CD11c^+^ DCs. **(A)** In the same donor, FCεR1α expression was compared between clones AER-37 and 9E1 for optimal expression, with corresponding Fluorescence Minus One (FMO) control. **(B)** Expression of defining conventional DC (cDC) 1 markers XCR1 and CD141 were compared in the same donor for correlation in intestinal tissue. **(C)** Representative plot of unstained intestinal cells, gated on events with higher side scatter. Autofluorescent negative and positive cells were gated and applied to un unused detector channel in FlowJo V10.7.1 to create the autofluorescent-corrected compensation matrix. **(D)** Representative plots for autofluorescent correction, showing correction of fluorescent signal for PEefluor610. **(E)** Representative plots of live HLA-DR^+^ CD45^+^ CD3^-^ CD19^-^ mononuclear phagocytes, comparing the CD11c BB515 signal with and without correction of autofluorescent signal. Autofluorescent macrophages overlaid, gated for CD14+ and autofluorescence.

With the ever-changing classification of DCs (11, 12, 14, 17, 18), their identification by flow cytometry has required an increasing number of discriminatory markers. For tissue DCs this task is further complicated by the cleavage of surface identification markers that often occurs during enzymatic liberation. A subset of cDC2 that express FCεR1α have recently been described (14, 18). We confirmed that FCεR1α was expressed on some cDC2s using the clone AER-37, though it was not until the same cells were stained with the clone 9E1 that we could confirm that the epitope recognised by AER-37 was being partially cleaved by collagenase type IV (**Figure 3A**). In addition to epitope cleavage, a further consideration is whether markers found on the surface of circulating cells correspond to tissue DCs. Blood cDC1s have been classified as CD141^+^ and XCR1^+^ (19), however, this does not appear to be the case in intestinal tissues where only a small portion of XCR1^+^ cells express CD141, the expression of which does not correlate to XCR1 expression (**Figure 3B**). Therefore, CD141 is not a reliable marker of cDC1s in the intestine. Taken together, these results emphasise the importance of marker and antibody clone selection for the proper identification of enzyme-liberated intestinal DCs.

### Autofluorescent Correction of Tissue-Resident Macrophage Subsets *via* Flow Cytometry

Tissue-resident yolk sac derived macrophages are characteristically autofluorescent (AF) (20). This unique characteristic allows for their identification by flow cytometry as AF CD14^+^ (13, 20). However, as more subsets of macrophages are defined in the intestine (10), their AF properties can mask the expression of key defining markers as well as presenting false positives. Roca, Burton (21) recently described a method for AF correction using AutoSpill, an algorithm for calculating spillover coefficients, by assigning AF as an additional endogenous dye using an unstained sample. We acquired an unstained sample of intestinal-liberated cells in addition to single-colour controls on beads to facilitate AF correction for manual compensation. Using the compensation wizard in FlowJo, the unstained sample was allocated to an unused detector channel where autofluorescence spills naturally, for example B710_50 on the FACSymphony, treating the AF signal from tissue cells as a single-colour control. By setting positive and negative gates for AF (**Figure 3C**) and re-calculating the compensation matrix with AF as a measurable parameter, AF spillover into other detectors was minimised, as shown with the correction of false signal using Fluorescence Minus One (FMO) (**Figure 3D**) and on CD45^+^ HLA-DR^+^ CD3^-^ CD19^-^ cells (**Figure 3E**). Furthermore, not using this method leads to inaccurate determination of surface marker expression as indicated for key DC expression marker CD11c in **Figure 3E**. Here we show that the CD14^+^ CD11c^-^ AF macrophages can be corrected to display as CD11c^-^, instead of spilling false signal into the detector allocated to CD11c.

### Identification of Intestinal-Derived Mononuclear Phagocytes

**Figure 4:**
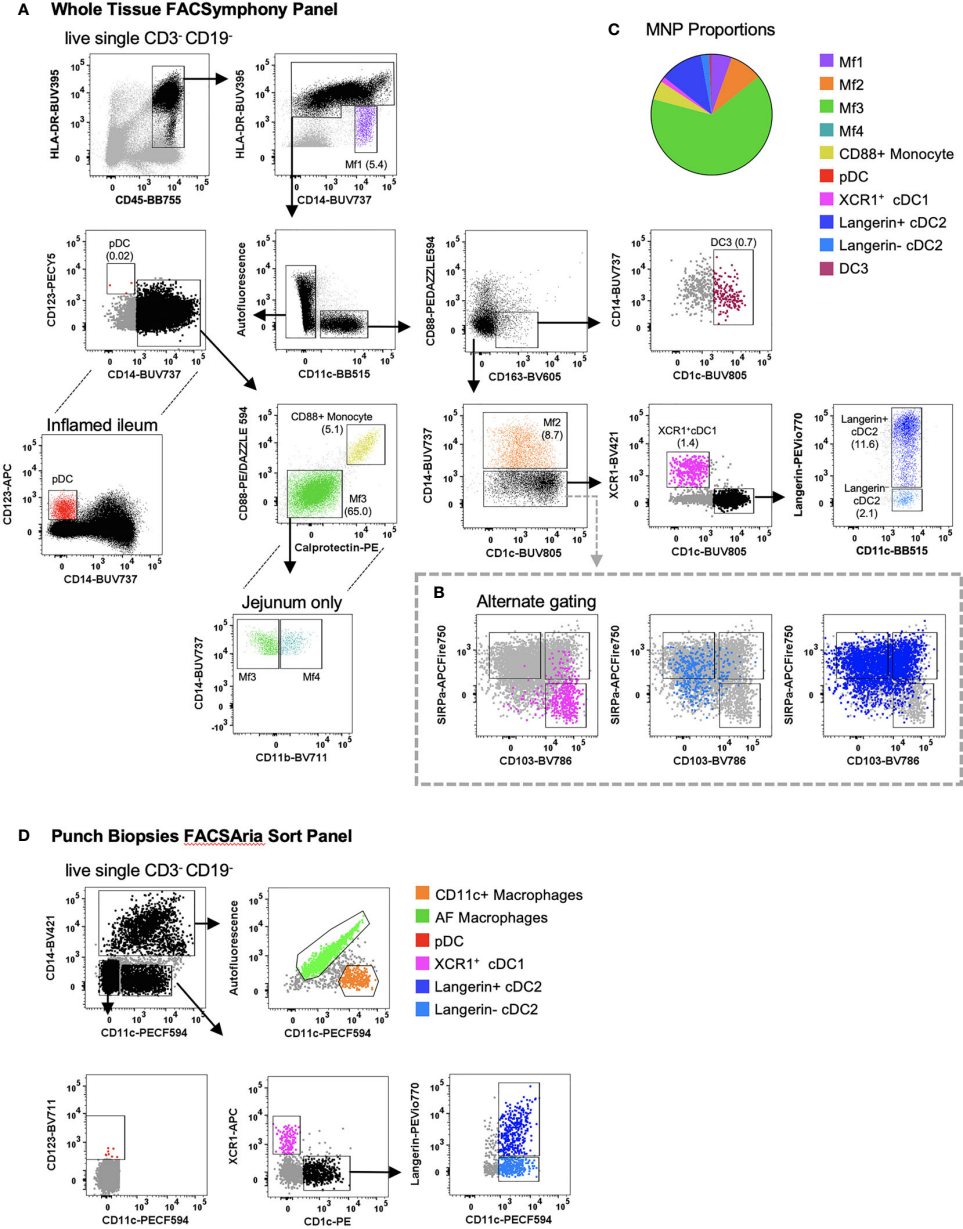
Identification of intestinal-derived mononuclear phagocytes by flow cytometry. Intestinal mononuclear phagocytes were isolated as per **Figure 1**. **(A)** Cells were stained with FACSymphony Phenotyping Panel (**Table 1**). All mononuclear phagocytes were gated within the live, single, CD45^+^ CD3^-^ CD19^-^ population. Intestinal mononuclear phagocytes (MNP) were gated in sequential order with percentage of total mononuclear phagocytes in brackets. Macrophage (Mf) 1 were defined as CD14^+^ HLA-DR^low^, with all sequential MNPs gated as HLA-DR^+^. Cells were then divided by their expression of CD11c. CD11c^-^ cells included CD14^-^ CD123^+^ plasmacytoid DCs (pDCs), CD14^+^ Calprotectin^+^ CD88^+^ monocytes and CD14^+^ autofluorescent^+^ Mf3s which could be further subdivided into CD11b^+^ Mf4 detectable only in the jejunum. CD11c^+^ cells were divided as follows: CD88^-^ CD163^+^ CD1c^+^ DC3s, CD14^+^ Mf2s, CD14^-^ CD1c^-^ XCR1^+^cDC1s, CD14^-^ XCR1^-^ CD1c^+^ cDC2s divided by their expression of langerin. **(B)** Alternative gating for CD11c^+^ CD14^-^ cells, characterised by SIRP*α* and CD103, overlaid with cDC1 and cDC2s from the main gating strategy for comparison. **(C)** Pie cart representation of proportion of mononuclear phagocyte subsets as part of whole of all mononuclear phagocytes **(D)** Cells isolated from intestinal biopsies were positively selected for CD45 and stained with FACS Sort Panel (**Table 2**). Live, single CD45^+^ HLADR^+^ cells were divided by CD14 expression. CD14^+^ cells were sorted as CD11c^-^ autofluorescent^+^ macrophages and CD11c^+^ macrophages. CD14^-^ cells were sorted as CD123^+^ plasmacytoid DCs, XCR1^+^ cDC1s, CD1c^+^ cDC2s langerin^+/-^.

MNPs share several common markers in tissue making their definitive identification and characterisation challenging (8, 10). Using flow cytometry, we were able to identify collagenase-liberated MNPs (CD45^+^ HLA-DR^+^ CD3^-^ CD19^-^) in intestinal tissue. We identified four subsets of CD14^-^ DCs: cDC1s expressing XCR1; two subsets of CD1c^+^ cDC2 divided by langerin expression; and CD123^+^ plasmacytoid DCs (pDCs) which, as expected, were more readily detectable in inflamed tissue (22) (**Figure 4A**). DC3s were identified as CD11c^+^ CD1c^+^ CD163^+^ CD88^-^ and CD14^+/-^ (14, 15). We also characterised intestinal DCs by their expression of CD103 and SIRPα (11, 12). The SIRPα^+^ DCs correlate with the langerin^+^ cDC2s, CD103^-^ DCs correlate to langerin^-^ cDC2s, and the SIRPα^-^ CD103^+^ cells correlate with the XCR1^+^ cDC1s (**Figure 4B**), showing that while there are distinct differences between human tissue DC phenotypes, there are overlapping similarities. In addition, CD14^+^ macrophages could be divided into four subsets: Mf1 (HLA-DR^low^), Mf2 (CD11c^+^), Mf3 (CD11c^-^) and Mf4 (CD11c^-^ CD11b^+^) (10), with Mf3 macrophages representing the largest proportion of mononuclear phagocytes (**Figure 4C**). We identified the Mf4 population in human jejunum (10), however have consistently been unable to identify these cells in lower intestinal tissues. Also identified were CD14^+^ CD88^+^ monocytes which can be further separated from macrophages by their expression of calprotectin (18). As whole tissue samples are not always abundantly available, we optimised a FACSort panel that would allow for the identification and isolation of MNPs from terminal ileum biopsies. As AF correction occurs post-acquisition, a gating strategy was devised to utilise AF spillover. We identified XCR1^+^ cDC1, langerin^+/-^ cDC2, pDCs as well as AF macrophages (Mf3) and CD11c^+^ macrophages (Mf1 and Mf2) for use in functional assays (**Figure 4D**).

### Mononuclear Phagocytes Are Phenotypically Immature at the Time of Liberation

**Figure 5:**
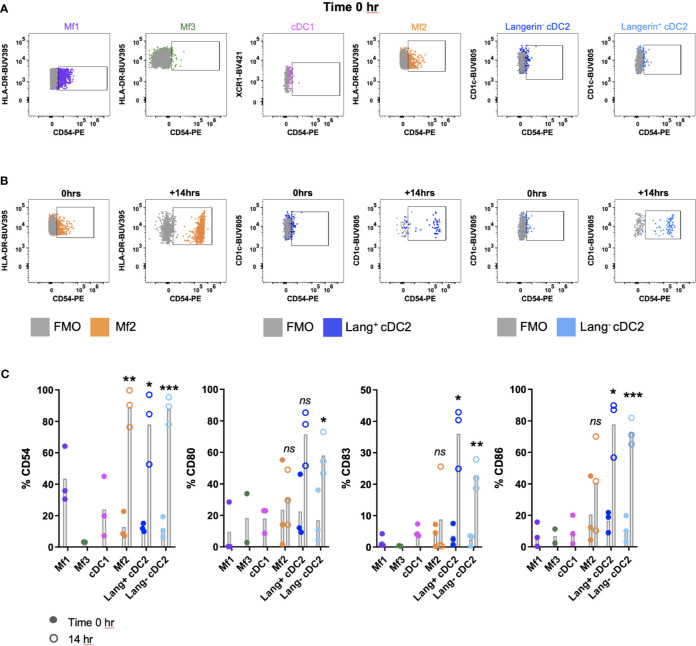
Investigating the maturation phenotype of tissue MNPs liberated by enzymatic digestion. Intestinal mononuclear phagocytes (MNP) were isolated as per **Figure 1**. Cells were stained for flow cytometry with FACSymphony Phenotyping Panel (**Table 1**) with drop-ins for CD54, CD80, CD83 and CD86, immediately following liberation (time 0 hr) as well as 14 hr post-liberation. **(A)** Cells were gated as per **Figure 4A**, with representative plots of time 0 hr expression of CD54 shown, compared to Fluorescence Minus One (FMO). **(B)** Representative plot for Mf2, langerin^+^ cDC2 and langerin^-^ cDC2 showing expression of CD54 at time 0 hr compared to 14 hr post-liberation, compared to FMO. **(C)** Expression of CD54, CD80, CD83 and CD86 on MNP subsets at time of liberation (closed circles), with 14-hr comparison (open circles) for Mf2, langerin^+^ cDC2 and langerin^-^ cDC2 (n=3). Statistics was by a paired Student’s t-test comparing each marker for each cell type at 0h with 14h (*p < 0.05, **p < 0.01, ***p < 0.001, ns, not significant).

A complication of the digestion process can be the inadvertent activation or maturation of cells (9, 23, 24). We investigated the maturation status of MNPs isolated by our digestion protocol, by assessing the surface expression of adhesion (CD54) and co-stimulatory molecules (CD80, CD83 and CD86), which are upregulated upon maturation (25). Immediately after isolation, all MNP subsets expressed low levels of CD54, CD80, CD83 and CD86 (**Figure 5A**). In two of three donors, Mf1s, Mf3s and cDC1s did not survive the 14-hour culture and therefore their maturation status at this time was not determined. However, significant upregulation of CD54 was seen on Mf2s, CD54, CD83 and CD86 on langerin^+/-^ cDC2s, with langerin^-^ cDC2 also upregulating CD80 post-culture (n=3) (**Figures 5B, C**). This culture-induced upregulation of maturation markers suggests that not only were the tissue-liberated MNPs viable, but they also remained immature throughout the enzymatic digestion.

### Liberated Intestinal Mononuclear Phagocytes Can Be Used for Functional Assays *Ex Vivo*

**Figure 6:**
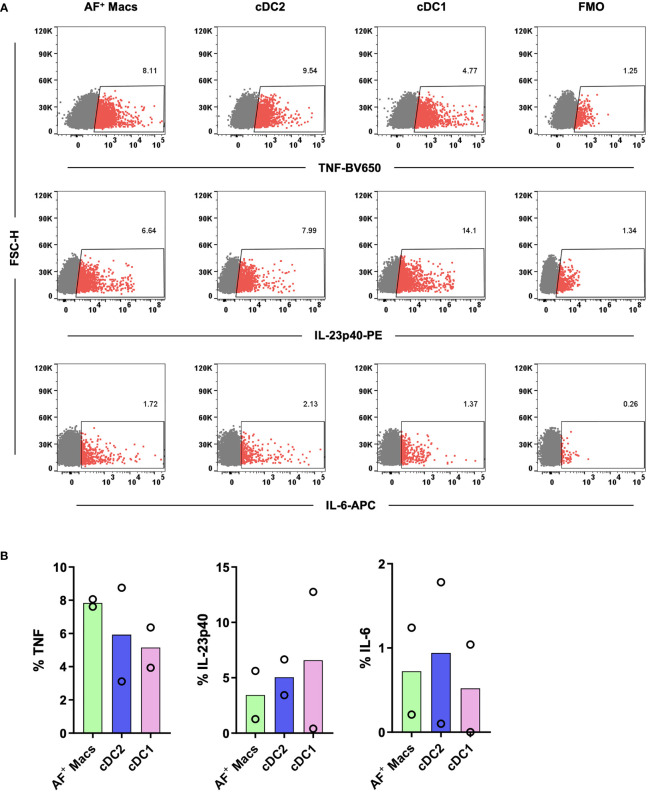
Investigation of the cytokine responses of tissue MNPs liberated by enzymatic digestion. Intestinal mononuclear phagocytes were isolated as per **Figure 1**. Cells were positively selected for CD45 and stained with FACS Sort Panel (**Table 2**). Autofluorescent (AF) macrophages (Macs), conventional DC (cDC) 1 and cDC2s were sorted based on gating in **Figure 4C**. Sorted cells were cultured at 1x10^6^ cells/mL in DC Culture Media with 1*µ*g/mL R848 for 2 hours. 2.5*µ*g/mL Brefeldin A was added, and the cells cultured for a further 14 hours. Cells were then stained with Fixable Viability Stain 700 and intracellularly stained with anti-TNF, anti-IL-23p40 and anti-IL-6. Cells were acquired on a FACSymphony. **(A)** Representative plots for expression of TNF, IL-23p40 and IL-6 on all subsets compared to Fluorescence Minus One (FMO). **(B)** Expression of TNF, IL-23p40 and IL-6 as a percentage of live single cells minus FMO, columns represent mean expression (n=2).

Having isolated and identified immature MNPs, we next confirmed that their biological functionality had been maintained. Sorted MNPs were stimulated and cultured for 16 hrs to determine their cytokine response. We observed, *via* intracellular staining, that AF macrophages, cDC1s and cDC2s could produce TNF, IL-23p40 and IL-6 in response to R848 (**Figure 6**). This cytokine response suggests that the intestinal-derived MNPs were liberated in a functional state.

## Discussion

The ability to interrogate immune cells from fresh human tissue is critical for advancing our understanding of the role these cells play in human disease settings. This is especially the case with intestinal MNPs, which are integral components of the mucosal innate immune system. Here, they play a crucial role in maintaining tolerance of the commensal microbiota while also remaining poised to respond to invading pathogens. While sophisticated animal models have greatly expanded our knowledge of intestinal MNPs, they are not able to completely recapitulate human tissue with many cells differing phenotypically and functionally (12, 26). Translating the data generated from these animal models requires the development of isolation methods to interrogate human immune cells *ex vivo* while maintaining their biological state. We previously described tissue digestion protocols for the isolation of MNPs from human abdominal skin (9) and anogenital tissues (8, 13). However, given the distinct phenotypic, functional and structural differences between skin and mucosal tissue, we present an isolation protocol to extract functionally immature MNPs from human intestinal tissue. We have carefully selected the optimal antibody clones and corrected AF spillover to develop high-parameter flow cytometry gating strategies to accurately identify all currently known subsets of MNPs in fresh human intestinal tissue.

An important consideration when isolating cells *via* enzymatic digestion is the delicate balance between cell yield and viability. Many groups perform one round of enzymatic digestion (10, 11), however, Lefrançois and Lycke (27) showed the advantages of performing a second round of digestion in murine mucosal tissues to enhance cell yield and viability. Our findings show that we can digest human intestinal tissue for a total of 90 minutes without compromising cell viability or proportions. As a result, we can liberate a higher quantity of total cells for downstream functional assays, which can be a limiting factor when working with human tissue-derived cells (13, 28). Most of the data we have presented here has taken advantage of our privileged access to whole pieces of intestinal tissue. While these larger tissue samples are often required to perform sorting for functional assays *ex vivo*, it is far more common to receive surgical biopsies from patients. To that end, we have shown that using our protocols it is possible to liberate, phenotype and even sort MNPs from human punch biopsies. Due to punch biopsies being smaller, a shorter digestion protocol is sufficient to liberate the cells which will make the interrogation of DC and macrophages from the intestine easier and far more accessible.

In the intestine, there are several different anatomical compartments containing varying ratios of functionally specialised immune cells each with their own unique interactions with the local intestinal environment (29). To interrogate these immunological compartments and the unique role each plays in disease settings it is crucial to isolate and interrogate them separately. The four primary compartments investigated in this study are the mucosal and submucosal layers, and their associated lymphoid follicles. Posing the greatest obstacle to deepening our knowledge of these immune compartments has been the difficultly in delicately separating the tissue layers and correctly identifying and removing the small lymphoid follicles. Other groups have attempted to expedite this task by using dyes to better visualise the lymphoid follicles (16), however, here we present an alternative method that removes this step and the risk it may pose in altering their function (30). Based on the method of Fenton, Jørgensen (16, 31), we were able to separate these compartments and show their unique immune profiles.

A known complication of enzymatic digestion is the cleavage of cell surface proteins, which can lead to the misidentification of cell subsets. We have previously shown that type IV collagenase best preserved the cell surface proteins used to identify tissue MNPs which led to the discovery of previously unidentified epidermal DC subset (8, 9). However, even with this collagenase blend, surface proteins can be partially cleaved, leading to underrepresentation of cell subsets within the tissue or their loss of function. It has proven particularly challenging to identify tissue-specific cDC2s as their markers overlap with monocytes and macrophages. Dutertre, Becht (18) revealed that FC*ϵ*R1*α* was one of two exclusive markers used to identify tissue cDC2s. Upon reviewing the literature, we selected FC*ϵ*R1*α* clone AER-37 as this was the most widely used clone. By using this specific antibody clone and type IV enzyme combination, we observed that FC*ϵ*R1*α* was partially sensitive to enzymatic cleavage as an unusually low proportion of cDC2s were FC*ϵ*R1*α* ^+^. It wasn’t until we selected a new FC*ϵ*R1*α* antibody clone: 9E1, that we were able to detect a much higher proportion of FC*ϵ*R1*α* expression on cDC2s in the intestine. Hence, FC*ϵ*R1*α* clone 9E1 with collagenase type IV is the superior combination.

Another consideration is the change in the pattern of marker expression on MNPs from different tissue compartments. In human blood and cutaneous abdominal tissue, cDC1s have been shown to express both CD141 and XCR1 (32). However, in human intestinal tissue we found that the expression of CD141 on cDC1s is extremely low and does not correlate with expression of XCR1. In addition, tissue cDC2s can upregulate their expression of CD141 (33). Peripheral blood cDC1s are defined by their CD141 and CADM1 expression, although CADM1 is not expressed by human skin cDC1s (9). We show here that CD141 is an unreliable marker in human intestine, therefore, we recommend XCR1 as the optimal marker for identifying cDC1, with the S154046E clone, in our experience as the only reproducibly reliable antibody that currently works on tissue-derived cells. This highlights the importance of carefully selecting antibodies that not only target preserved receptor epitopes post-digestion but reflect the expression of cell surface receptors in the tissue of interest.

The classification of MNPs is a rapidly evolving field of research and identifying them by flow cytometry can be challenging as subsets of MNPs share common markers (8, 10). This is further complicated by the AF properties of tissue-resident macrophages. Here we were able to correct AF by using an unstained tissue-derived control sample, allowing us to differentiate between real and false signal and improve resolution of surface markers used to identify MNPs. We could identify all currently known subsets of intestinal DCs using CD103 and SIRP*α* (11, 12) as well as the newly published DC3s (14, 15) in the same tissue. Furthermore, the SIRP*α* ^-^ CD103^+^ cells could be identified as XCR1^+^ cDC1 and SIRP*α* ^+^ CD103^+/-^ cells as CD1c^+^ (langerin^+/-^) cDC2, which is more relevant across different tissue types.

Following enzymatic extraction of MNPs from human intestinal tissue we have optimised methods to interrogate the function of these cells. This is important as extraction of MNPs from the tissue by enzymatic digestion can alter their biological function. Mature tissue MNPs have been shown to express elevated levels of leukocyte adhesion molecule CD54 (ICAM-1) and co-stimulatory molecules such as CD80, CD83 and CD86 (25). Conversely, immature MNPs freshly isolated from healthy human intestinal tissue should express low levels of these receptors on their surface. Following overnight culture, we showed that Mf2s and cDC2s had a substantial increase in maturation marker expression confirming that the cells were in an immature state upon liberation. Interestingly, we observed that Mf2s and cDC2s survived the culture, whereas Mf1s, Mf3s and cDC1 did not survive in two out of three donors. This suggests that cDC2 and Mf2, which are phenotypically very similar (10), share a propensity for survival outside of their tissue environment. We also showed that tissue liberated MNPs could produce cytokines in response to a TLR7/8 ligand. These findings confirm that intestinal MNPs liberated by enzymatic digestion following our optimised mucosal tissue digestion protocol are in an immature and biologically functional state. This is advantageous over other isolation methods such as spontaneous migration of DCs out of tissue during culture, which triggers the maturation of DCs limiting their use in functional assays (9).

In summary, our study demonstrates an enzymatic digestion protocol for the isolation of human intestinal MNPs in an immature state from tissue and biopsies while maintaining their biological function. We provide a method for the anatomical separation of the different gut-associated lymphoid tissues without the use of dyes. We also demonstrate the need for careful selection of antibodies that target preserved receptor epitopes and reflect their unique tissue-specific phenotype. These optimised protocols will greatly enhance our ability to interrogate APCs in human tissue, which will be important to understanding their role in homeostasis and diseases of the intestine.

## Data Availability Statement

The raw data supporting the conclusions of this article will be made available by the authors, without undue reservation.

## Ethics Statement

The studies involving human participants were reviewed and approved by Western Sydney Local Area Health District (WSLHD) Human Research Ethics Committee (HREC); reference number (4192) AU RED HREC/15 WMEAD/11. The patients/participants provided their written informed consent to participate in this study.

## Author Contributions

CD and EV helped design and performed most of the experiments, analysed the data, prepared the figures and wrote the first draft of the manuscript. KB, HB and JR helped design, interpret and perform some of the experiments. MG, AD, GC, FR, JT, NP-N and GA provided the human tissue samples and intellectual input. GC and AC helped design the experiments and interpret the data. AH and SB supervised CD and EV, designed the experiments, analysed and interpreted the data, finalised the figures and wrote the final draft of the manuscript. All authors contributed to the article and approved the submitted version.

## Funding

This work was supported by the Neil and Norma Hill Foundation, the Westmead Medical Research Foundation and the Australian National Health and Medical Research Council (NHMRC Project Grant #APP1078697, Ideas Grant #APP1181482).

## Conflict of Interest

The authors declare that the research was conducted in the absence of any commercial or financial relationships that could be construed as a potential conflict of interest.

## Publisher’s Note

All claims expressed in this article are solely those of the authors and do not necessarily represent those of their affiliated organizations, or those of the publisher, the editors and the reviewers. Any product that may be evaluated in this article, or claim that may be made by its manufacturer, is not guaranteed or endorsed by the publisher.
